# Optimal Design of Water Quality Monitoring Networks in Semi-Enclosed Estuaries

**DOI:** 10.3390/s20051498

**Published:** 2020-03-09

**Authors:** Nam-Hoon Kim, Jin Hwan Hwang

**Affiliations:** 1 Marine Disaster Research Center, Korea Institute of Ocean Science & Technology, Busan 49111, Korea; nhkim426@gmail.com; 2 Institute of Construction and Environmental Engineering, Seoul National University, Seoul 08826, Korea

**Keywords:** optimal design procedure, monitoring network, water quality, graphical optimization, objective mapping

## Abstract

The semi-enclosed estuary is very susceptible to changes in the physical and environmental characteristics of the inflow from the land. Therefore, continuous and comprehensive monitoring of such changes is necessary for managing the estuary. Nevertheless, the procedure or framework has not been proposed appropriately to determine how many instruments are necessary and where they need to be monitored and standardized to detect critical changes. The present work proposes a systematical strategy for the deployments of the monitoring array by using the combination of graphical optimization with the objective mapping technique. In order to reflect the spatiotemporal characteristics of the bay, the representative variables and eigenvectors were determined by the Empirical Orthogonal Function (EOF), and the cosine angle among them calculated and used as a design index of optimization. At the recommended locations, the sampled representative variables were interpolated to reconstruct their spatiotemporal distribution and compared with the true distribution. The analysis confirmed that the selected locations, even with a minimal number of points, can be used for on-site monitoring. In addition, the present framework suggests how to determine installable regions for real-time monitoring stations, which reflect the global and local characteristics of the semi-enclosed estuary.

## 1. Introduction

An estuary is a coastal area where seawater from offshore meets freshwater from rivers and is dominated by various control sources such as wind, solar radiations, tidal strength, river discharge, bathymetry, etc. In particular, river discharge is a source of nutrients for estuaries, but can sometimes have a negative impact due to releasing contaminants from upstream together. Moreover, due to the fast urban-sprawl or urbanization near coastal areas, the amounts of released pollutants are increasing vigorously and flowing into coastal seas through the river from urbanized watershed. The coastal areas influenced by freshwater are often semi-enclosed, hence, once contaminants originating from the watershed flow into the bay, they can accumulate and continuously deteriorate water quality [1,2,3]. Along with the deterioration of the water environment, the demands for water supply have soared, ultimately leading to the request for the construction of many sea-dikes for the coastal reservoir to secure the water resources. However, such sea-dikes efficiently inhibit the tidal momentum of the offshore sea from advecting to the river and near coastal waters, and, therefore, significantly affects the physical characteristics of the water column, such as the stratification, mixing, and circulation of flow [4,5,6]. In addition, the gates of the sea-dikes are discharging freshwater irregularly to maintain the water level of the upstream to be constant, hence the physical characteristics and water quality of the coastal sea change complicatedly and unexpectedly [7,8,9].

The estuaries of the West Sea of Korea are shallow, with wide tidal-flats, considerable tidal variations of the macro level, and complex geomorphology. Moreover, they are exposed to the physical and environmental alterations caused by the irregular discharges of freshwater from the gates of the coastal reservoir [7,10,11]. Inflows from the watershed lead to unexpected imbalances in the nutrients and red tides, caused by eutrophication, often occur in the summer (i.e., flood season) [12,13]. Sometimes, water quality worsens due to the leakage of the green algae from the upstream of the reservoir [14]. Furthermore, a decrease in numbers of freshwater discharge in the winter due to low rainfall (i.e., dry season) can severely affect salt-sensitive aquacultures [15,16]. Therefore, an earlier detection of changes in the nutrients, water temperature, and salinity are essential to mitigate the impact in advance, understand the natural process, and manage the environment soundly.

Generally, two approaches have been mainly used to monitor the coastal sea: on-site monitoring and continuous real-time monitoring [17]. The on-site monitoring is a way of visiting the site of interest, carrying instruments regularly to monitor the characteristics of the bay. The continuous real-time monitoring is a way of collecting data remotely by installing the unmanned observatory. In any case, an important question can be raised as to how many and where the sensors or stations should be installed to sufficiently represent the spatial and temporal characteristics of the region of interest. Therefore, strategies need to be established and standardized to deploy and operate the monitoring array for managing sound environments. Even though several strategies have been proposed previously, such as the guidelines of the United States Environmental Protection Agency (US EPA) [18], the monitoring locations have been judged arbitrarily by the discussions of stakeholders, engineers, and decision-makers rather than based on robust and reliable systematic protocol or design guidelines [17,19,20]. Therefore, the strategies for deploying and operating a monitoring network need to be provided for the sound management of the coastal and ocean environment since the scientifically solid and robust data are essential in preparing countermeasures for decision making [17].

In the design of the monitoring locations, some requirements should be specified. Since the variables of interest are best measured simultaneously over the whole domain within a predetermined time, limited numbers of measuring points should be optimally selected, which still must be sufficient to represent the spatiotemporal characteristics of the target region. Several prior studies have been carried out to meet these requirements. In [21], the authors conducted a trial-and-error method to find the points to best reconstruct the so-called objective mapping for visualizing the data. After the introduction of the objective mapping technique, some studies have followed, focusing mainly on mapping-based optimization, which can compensate for the limitations of the trial-and-error method (e.g., [22,23,24]). Such prior studies designed arrays that can best reconstruct the spatial distribution by applying the optimization technique, such as the simulated annealing [22] and genetic algorithm [23] to minimize the covariance function or spatial averaged quadratic error [24]. Furthermore, many types of research have been performed to find the best objective mapping for the applications in diverse fields, such as mooring locations to measure the sea level altitudes [25], sensor arrays to monitor the oceanic meridional overturning circulation [26], and the collection data for the modeling with the data assimilation [27,28].

Such developments recently led to the redesign of the existing monitoring network, that had been intuitively and arbitrarily designed in the past. For example, [29] constructed the objective function based on the principal component analysis and solved it with spatial sampling optimization to eliminate redundant points in the Yangtze River Estuary, China. In addition, [30] and [31] performed a similar study using the Kriging and spatially simulated annealing method in the Changjiang Estuary and Hangzhou Bay, China, respectively. Most recently, [19], which is a work that set the precedent for the present study, also proposed a primitive version of the framework designing the monitoring system using a similar method to the previously researched.

Most of the prior studies performed analyses mainly on the ideal case (e.g., [21,22,23]) or for the large-scale ocean (>5000 km) (e.g., [24,26]) rather than small-scale waters (<50 km) such as the coastal bays or estuaries, except [19]. In general, the spatial and temporal variabilities of hydrodynamic and water quality variables on the global- or large-scale seem to follow a more natural variation. However, the coastal water must depend not only on the global- or large-scale variability, but also significantly on the process of the land through the river. Therefore, the spatial variability of the characteristics in the coastal domain cannot be adequately represented by the same technique for the large-scale area. In addition, most of the prior studies did not design an integrated monitoring network which can detect the diverse variables simultaneously, but focused on a single variable, such as current [22,23,26], salinity [32], or water quality variables [29,30,31] to find the design variable for optimization.

As a recent study, [33] used the objective function as the quantitative function (i.e., scalar function), such as Root-Mean-Square-Error (RMSE) or covariance, etc. They found that such a quantitative objective function not only requires a long computation time to find the solutions, but that it is also hard to prevent the results from falling into the local solutions. Furthermore, the optimization techniques based on the quantitative objective function are only suitable for the solutions of “how many” points are to be arranged, and it is hard to find the solutions to “where” they are to be located. Moreover, there is little research to determine the locations of the real-time monitoring station [34,35,36].

Therefore, the present study is to propose a well-organized framework for designing a water quality monitoring network in the small-scale estuarine area. In order to reconstruct the spatiotemporal distribution to represent the variabilities of the target variables in the small-scale area, a graphical optimization technique is applied to find the best locations for the representative monitoring array by constructing the objective function of the optimal mapping approach. Since the graphical optimization technique can directly select the arrays of monitoring points in the continuous field, the computation time is short, and there is no possibility of falling into the local problems. Moreover, this technique is excellent in its application to the problems of the steep gradient of signals with significant spatial variations due to the freshwater discharges. We present the methodologies for setting up the experiment in Section 2, the results and discussion of the design for a water quality monitoring network based on the scenarios in Section 3, and the conclusions in Section 4.

## 2. Materials and Methods

### 2.1. Characteristics of the Study Area

**Figure 1:**
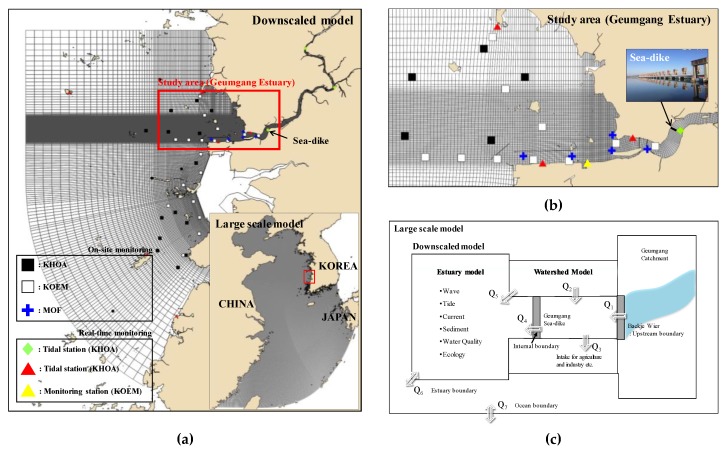
Monitoring status and computation grid of the (**a**) large scale model (117.88˚ E–131.36˚ E; 23.92˚ N–41.15˚ N) and downscaled model (125.85˚ E–127.01˚ E; 35.19˚ N–36.33˚ N), (**b**) Geumgang Estuary (126.3˚ E–126.8˚ E; 35.9˚ N–36.2˚ N) and position of the sea-dike (126.75˚ E; 36.02˚ N), and (**c**) concept of the integrated modeling. The abbreviations of KHOA, KOEM, and MOF imply Korea Hydrographic and Oceanographic Agency, Korea Marine Environment Management Corporation, Ministry of Oceans and Fisheries, respectively.

The Geumgang Estuary (hereafter GE) is located at the west coast of Korea (Figure 1) and categorized as a well-mixed estuary, where a semidiurnal tide dominates [5]. The sea-dike is located in the mouth of the Geumgang river, which is one of the main rivers in Korea, and GE refers to the sea area about 55 km in the *x*-direction and 35 km in the y-direction from the sea-dike (Figure 1b). GE has substantial variabilities of salinity since the freshwater is released irregularly and artificially from the coastal reservoir [37]. The amount of artificially discharged freshwater depends on the water level of the reservoir, which is closely related to the rainfall on the upstream watershed (Table A1). The amount of the discharged freshwater also determines the physical and environmental characteristics of the coastal seawater, such as water temperature and salinity, along with the concentrations of dissolved materials (e.g., nitrogen, phosphorus, chlorophyll, dissolved oxygen, etc.) and so significant changes in freshwater discharge cause large variations in the water quality. In [19], the authors conducted an on-site observation for three years using the Conductivity, Temperature, Depth (CTD) sensor, the multi-parameter water quality sensor, and water sampling at GE to acquire water temperature, salinity, and water quality variables (i.e., dissolved oxygen, chlorophyll-a, total nitrogen, and total phosphorus). They found that measured variables are controlled by the amount of rainfall (i.e., the main factor of freshwater discharge) and seasonal variability. Specifically, water temperature moves together with dissolved oxygen, total phosphorus, and chlorophyll-a and salinity with total nitrogen. Moreover, [4,5] performed line measurements, suggesting that the physical characteristics (e.g., mixing and stratification) have seasonal variability depending on the frequency of rainfall, and that the intrinsic characteristics could change. There needs to be an advanced awareness of such changes in the water quality, which occur mainly due to freshwater discharge, to sustain the sound environmental conditions, but the monitoring points of the GE are currently sporadically arranged without any specific guidelines (Figure 1b). In particular, the on-site monitoring points are randomly arranged, and there is only one real-time water quality monitoring station (yellow triangle). Since the monitoring network of GE was arbitrarily designed, GE may be monitored irrationally at the moment. Therefore, it is somewhat difficult to analyze and find intrinsic characteristics of the GE, where it is strongly affected by the freshwater discharged from the upstream. Therefore, well-organized guidelines for the monitoring network design are necessary. 

### 2.2. Numerical Model (Input Data)

Seamless spatiotemporal information should be used as input data to design a monitoring network appropriately, but it is hard and expensive to perform field measurements for a long enough duration and in large enough areas. Because of this, the scattered data of field measurements have limitations in their direct use for the design, since they are available only at several specific points and during certain periods. An alternative approach could be to use the data from the satellite images instead, but they depend too much on the daily weather. Therefore, it is tough to obtain continuous spatiotemporal information, hence using the satellite data can potentially miss some critical points and certain periods. For these reasons, the present work hired sets of the spatiotemporally highly resolved and well-validated numerical simulation data. The advantage of using the results from the numerical simulations is that diverse physical and environmental variables can be extracted from the numerical model simultaneously, which can be considered together as input data for designing the monitoring network.

This study assumed that the numerical model results simulated by [15] are real data to design the monitoring network. Figure 1 shows a conceptual diagram, domain, and grid of the numerical model. A three-dimensional hydrodynamic model of Delft-3D [38] simulated the hydrodynamics and water quality near the coast. The initial and boundary conditions were carefully downscaled from the large-scale model of the Yellow Sea regional model (Figure 1a) [15]. The numerical model was simulated for two years from January 2014 to December 2015 and the model results, corresponding to about 55 km in the *x*-direction and about 35 km in the *y*-direction (i.e., GE), were extracted to apply and analyze (Figure 1b). In the numerical simulation, the initial and boundary conditions of freshwater discharge from the upstream were generated by a watershed model, STREAM [15]. This model is a squared uniform grid and quasi-distributed watershed model that can simulate flow, sediment, and water quality of the watershed (Figure 1c). 

The simulation results were calibrated and validated for each variable using data measured directly by the author during the same period as the simulation period, and data obtained from monitoring points shown in Figure 1a operated by various organizations (see more detail in [15,19]). Moreover, the accuracy of the model results has been improved with the calibration and validation step for each variable using the Index of Agreement (IOA) [39] and Relative Error (RE) [40], respectively. Overall, even though the water quality variables have slightly lower skill scores than the hydrodynamic, both variables still have strong correlations with the observation data (Table A2).

### 2.3. Design Variables

Six variables were selected for analysis and applied as depth-averaged values; water temperature (T), salinity (S), dissolved oxygen (DO), chlorophyll-a (Chl-a), total nitrogen (TN), and total phosphorus (TP), which can commonly be obtained from the real field monitoring. The reason for considering multiple variables is to select representative variables among them to design the monitoring network. If the optimal location is determined by the representative variables, and other variables with high reliability are detected at that location, then we do not need to design the monitoring network complicatedly considering all the other variables. Therefore, it is imperative to select variables that can reflect the characteristics of other variables as a design variable. To reduce the number of variables and find a variable representing others, we used the Empirical Orthogonal Function (hereafter EOF) to compress an extensive data set into a smaller number of independent pieces of information [41,42], since it is hard and expensive to determine the convergence threshold of the objective function for each variable.

As the first step of the EOF, the eigenvalues corresponding to the series of a linear system need to be found, which can be expressed as follows:

(1) Cϕ−λIϕ=0,

where the covariance matrix, C, consists of *M* elements of the data with the length of *N* (*M*×*N*). I is the unity matrix, and ϕ is the EOF. The EOF, corresponding to the eigenvalue $\lambda_{M}$, is the uncorrelated (i.e., orthogonal) mode of variability. If equation (1) is to have a nontrivial solution, the determinant of the coefficients must vanish and yield an *M*th order polynomial, $\lambda^{M}+\alpha \lambda^{M-1}+\cdots$, whose *M* eigenvalues satisfy $\lambda_{1}>\lambda_{2}>\cdots >\lambda_{M}$ [42]. Thus, the variances associated with each statistical mode are ordered according to their corresponding eigenvectors. The first mode, $\lambda_{1}$ contains the highest percentage of the total variance, and among the remaining variances, the greatest percentage is in the second mode, $\lambda_{2}$, and so on [42]. This method can reduce the information of each variable to represent the variance concerning the eigenvectors.

**Figure A1:**
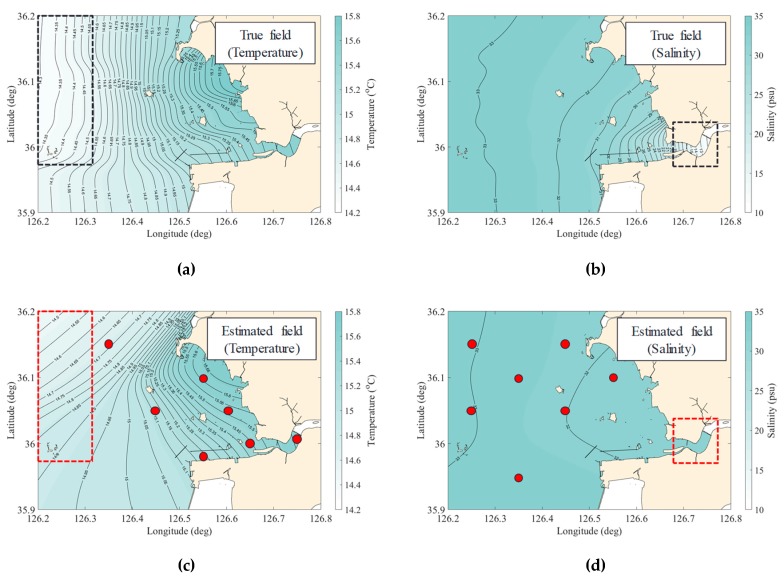
The true field of (**a**) water temperature and (**b**) salinity, and the example of the monitoring array (**c**) designed by considering only salinity and reconstructing the spatial distribution of the water temperature, and (**d**) designed by considering only water temperature and reconstructing the spatial distribution of the salinity.

The present work also chose a cosine angle between two eigenvectors of the representative variables in the three-dimensional Euclidean principal component (hereafter PC) space as a design variable for constructing the monitoring network. PC is constructed by the normalized six variables in this work, and two eigenvectors refer to the two most independent variables among six variables of T, S, DO, Chl-a, TN, and TP. The reason we selected a slightly complicated index as a design index is that if one variable is chosen for a design variable, other variables are hard to monitor appropriately since each variable could have different spatiotemporal variabilities due to their different sources. For example, water temperature is mainly determined by the local solar radiation and also, water temperature from the open sea. However, salinity is mainly determined locally by the amount of the freshwater discharge from the river, so if the monitoring array is designed solely by salinity, this designed array is not likely adequate to detect the variations of water temperature (Figure A1a,A1c). Conversely, when the monitoring array is designed only by water temperature, the reconstructed distribution of salinity is totally different from the true distribution (Figure A1b,A1d).

Therefore, since the cosine angle can represent the characteristics of variables with different origins, the use of it allows for considering several variables simultaneously by the monitoring networks. The cosine angle between two vectors can be expressed as follows:

(2) $$\cos\left(a,b\right)=\frac{a\cdot b}{\left\|a\right\|\cdot \left\|b\right\|}=\frac{\sum_{i=1}^{n}a_{i}b_{i}}{{\left\{\left(\sum_{i=1}^{n}a_{i}a_{i}\right)\times \left(\sum_{i=1}^{n}b_{i}b_{i}\right)\right\}}^{1/2}},$$

where $a=\left(a_{1},a_{2},\ldots ,a_{n}\right)$ and $b=\left(b_{1},b_{2},\ldots ,b_{n}\right)$ are two eigenvectors of the representative variables. In the three-dimensional Euclidean PC space, *n* must be three.

### 2.4. Finding the Optimal Solutions

Once the design variable is determined, the optimization is performed to find solutions of the most appropriate numbers and locations for the monitoring in the domain of target. The general optimization problem is posed as follows:

(3) $$\begin{array}{ll} \mathrm{Minimize} & \;f\left(X\right)\\ \mathrm{subject}\;\mathrm{to} & \;g_{i}\left(X\right)\leq 0\;\left(i=1,2,\ldots ,m\right);\\ & \;h_{j}\left(X\right)=0\;\left(j=1,2,\ldots ,p\right);\\ & \;X^{lower}\leq X\leq X^{upper}; \end{array}$$

where f(X) is the objective function; $g_{i}\left(X\right)$ is the *i*th inequality constraint; *m* is the total number of inequality constraint functions; $h_{j}\left(X\right)$ is the *j*th equality constraint; *p* is the total number of equality constraints; X is the vector of design variables; and $X^{lower}$ and $X^{upper}$ are the lower and upper bounds of the design variables, respectively. To find the optimal solutions in a constrained optimization problem, it is necessary to construct feasible regions reflecting various constraint violations. Thus, the constrained optimization problem needs to be transformed into the unconstrained optimization problem by adding penalty terms for each constraint violation [19,43,44,45,46]. Finally, after transforming, the objective function (i.e., augmented function) is solved by heuristic optimization, such as a genetic algorithm. This procedure is called an Augmented Lagrangian Genetic Algorithm (ALGA), which finds a set of stable solutions satisfying the Kuhn–Tucker conditions by mathematically handling a large number of constraint functions with less computational cost [46,47].

To find the optimal solutions, we employed two optimization problems for comparison; one was a quantitative approach, and the other a graphical approach. The quantitative optimization finds an optimal arrangement, which can reconstruct the spatiotemporal distribution by constructing a quantitative objective function of the root-mean-square error (RMSE), which is expressed as follows:

(4) $$f\left(X\right)=\sqrt{\frac{1}{N}\sum_{i=1}^{N}E_{i}-T_{i}},$$

where $E_{i}=\left(E_{1},E_{2},\ldots ,E_{N}\right)$ and $T=\left(T_{1},X_{2},\ldots ,T_{N}\right)$ are the estimated and true values, respectively. The quantitative objective function, such as RMSE, requires a long computation time to find a solution, and its result could easily fall into the location solution, hence there is no means to prove whether a given solution is the best. Therefore, even though this method has been applied to the selections of numbers of the observation points [32], the different arrays are possible to determine for each iteration due to arbitrary array selection of the locations.

The graphical optimization constructs the primary function of the optimal interpolation as an objective function and can find the optimal arrangement that represents the continuous spatiotemporal distribution [19]. Currently, the most widely used optimal interpolation schemes in meteorological and oceanographic applications may be the statistical interpolation, also known as the Optimal Interpolation (OI) scheme [48], or the Barnes Objective Analysis (BOA) [49]. Even though the OI is most prevalent to estimate the ocean data field (e.g., [21,50,51,52,53]), it is not ideal to use in this study since the assumptions of spatial homogeneity and isotropy are not relevant to a small (≤50 km) and highly dynamic area such as the small coastal seas.

The objective analysis is often referred to as a process of transforming data from observations at irregularly spaced points into data at the points on a regular space grid [54] for meteorological purposes. In [49], the authors modified this scheme to interpolate the whole complex region of interest by repeatedly applying a distance-dependent weighting [55,56,57]. The objective function of the graphical optimization for designing the array of the monitoring points can be constructed by the loop function of BOA and can be expressed as follows:

(5) $$f\left(X\right)=\pm \left[\frac{\sum_{m=1}^{N}w_{m}R\left(X_{m}\right)}{\sum_{m=1}^{N}w_{m}}+\frac{\sum_{m=1}^{N}w_{m}^{\prime }\left(2R\left(X_{m}\right)-E^{1}\left(X_{m}\right)-E^{2}\left(X_{m}\right)\right)}{\sum_{m=1}^{N}w_{m}^{’}}\right],$$

where $R\left(X_{m}\right)$ is the reference value (i.e., design variable) at the location *m,* and $E^{1,2}\left(X_{m}\right)$ are the estimates at each loop extracted at the same location to the reference value. The *m*th weights are as expressed in equations (6) and (7):

(6) $$w_{m}=\exp\left[-\left(\frac{d_{mx}^{2}}{c_{x}^{2}}+\frac{d_{my}^{2}}{c_{y}^{2}}\right)\right],$$

(7) $$w_{m}^{\prime }=\exp\left[-\left(\frac{d_{mx}^{2}}{\gamma c_{x}^{2}}+\frac{d_{my}^{2}}{\gamma c_{y}^{2}}\right)\right],$$

where $d_{m}$ is the distance between the grid point and the *m*th reference point, and the length scales $c_{x}$ and $c_{y}$ control the fall-off rate of the weighting function in the different rates to *x* and *y* directions [55,56,58]. The length scale could be solved by a nonlinear curve-fitting method of the Levenberg–Marquardt least square method [59]. γ is a numerical convergence parameter that controls the difference between the weights on each step for the range of 0 to 1 [60].

In the graphical optimization, the objective function of Equation (5) constructs the spatial distribution (i.e., domain) of the design variables to describe the target bay. The elliptic radius weighting function of Equation (6) is the distance from the center to the border of an area described by the constrained function, which keeps the solution to be out of the radius of influence while finding the optimal solution. In addition, land and structures can be composed of several exterior nonlinear and graphical functions [19]. Therefore, the solutions of maximum and minimum are located inside of the ellipse constraints (i.e., feasible region), refraining geomorphology from constraining them. To construct the objective function, BOA is selected due to its simplicity and applicability to the wide ranges of scales. It is also suitable for use in conjunction with the graphical optimization technique.

### 2.5. Methods of Performance Evaluation

Once the optimal solutions (i.e., monitoring array) are found, the spatiotemporal distribution of the variables is reconstructed with the solutions, and validated by comparing them to the original data. The skills of the reconstruction can be evaluated by the statistical metrics [61], which tell the difference between true and estimated values. The present work used two types of skill metrics; the Taylor diagram [62] and the target diagram [63]. These diagrams compile the statistical measures of the reconstruction skill into a single graph to allow for the comparison and analysis of the various cases. The Taylor diagram graphically summarizes and compares two sets of results regarding three statistics: correlation coefficient (COR), standard deviation (SD) of the true (subscript *T*) and estimated (subscription *E*) fields, and centered (i.e., unbiased) root mean square difference (CRMSD), which have the following relationship:

(8) $$CRMSD^{2}=SD_{T}^{2}+SD_{E}^{2}-2\times SD_{T}\times SD_{E}\times COR.$$

Another tool to evaluate the skills is a target diagram, which is derived from the relationship between the metrics of Bias, which means the difference of the mean values, *CRMSD*, and *RMSD*. This diagram used a Cartesian coordinate system where the *x*-axis represents the *CRMSD*, the *y*-axis represents the *Bias*, and the diagonal distance (radius) indicates the *RMSD*. *CRMSD* is an unbiased *RMSD* and removes any potentially biased information [63]. The following relationship relates these three statistics:

(9) $$RMSD^{2}=Bias^{2}+CRMSD^{2}$$

## 3. Results and Discussion

### 3.1. Decomposition of the Spatiotemporally Dependent Variable

**Figure A2:**
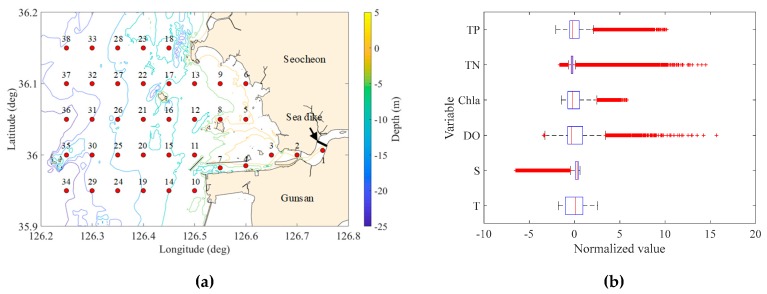
(**a**) The initial placement of the monitoring points and (**b**) boxplot of each variable.

As the first step to find the design variable, the initial monitoring points were distributed over the entire domain (Figure A2), and the time-series of each variable were extracted at those points from the numerical model. In order to avoid dimensional heterogeneity, each variable was subtracted from the mean values of each variable, and we divided those differences by the standard deviations to obtain normalized values to represent each variable (Figure A2).

**Figure 2:**
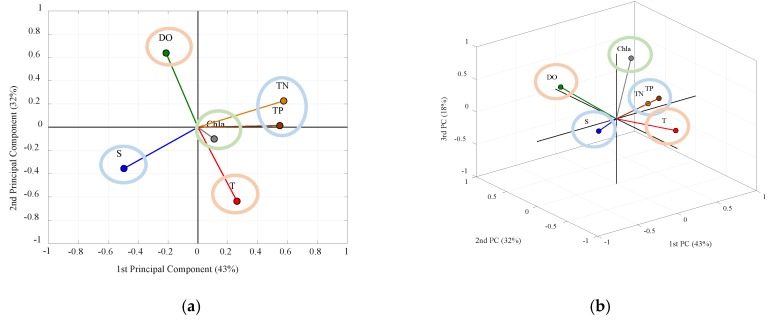
The results of the empirical orthogonal function (EOF) corresponding to the (**a**) 2D and (**b**) 3D principal components (PCs) for the spatial distribution. The sky blue, orange, and light green mean a group of variables that contribute to the first PC, second PC, and third PC, respectively.

The most representative ones among the given variables were selected by the EOF analysis of the spatial distributions of the normalized six variables. Figure 2 shows the eigenvectors of the six variables, and Table 1 summarizes the results of the EOF analysis in detail. When the eigenvalues from the EOF are smaller than 1, those values are not significant, and so we used the first three among the six PCs for which the eigenvalues were larger than 1 [64]. The vectors in Figure 2 are constructed with the PCs representing the distribution of each variable. For example, the eigenvector representing salinity is composed of −0.5 of the first component, −0.36 of the second one, and 0.15 of the third one (Figure 2 and Table 1). In Figure 2, the *x*-axis represents the contribution of the variable to the first PC, the *y*-axis represents that for the second PC, and *z*-axis does for the third PC. When the magnitudes of the eigenvector are smaller than 0.5, the variables represented by that eigenvector are not significant [64], and so we do not consider them in the analysis.

Once the eigenvectors for each variable are calculated, six variables are categorized in the three groups depending on which PCs they contribute to. Among six variables, TN, TP, and S contribute to the first PC, which is 43% of the total variances, and T and DO contribute to the second PC of 32% (Figure 2a and Table 1). Chl-a mainly contributes to the third PC, but only by 18% of the total variances, hence was excluded from consideration (Figure 2b and Table 1). Therefore, the two groups contributing to the first and second PC are independent of each other since all PCs are orthogonal to each other. Of all the variables in the two groups, S and T each contribute to the first and second PCs with a relatively larger magnitude than the other variables. Furthermore, they are also usually measured by one instrument simultaneously. Therefore, T and S were selected as representative variables to construct an index.

**Figure 3:**
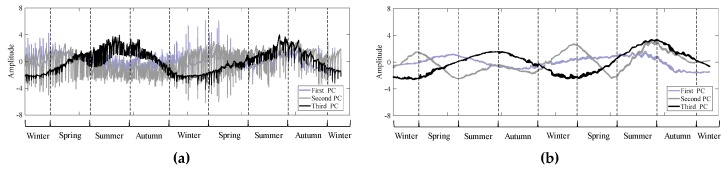
First, second, and third PC time-series of six decomposed variables extracted from (**a**) pt. 1 (near the sea-dike) and (**b**) pt. 38 (ocean side).

Once selecting the two most representative variables from the spatial PC analysis, the variables were analyzed again by the temporal EOF. Figure 3 shows an example of the time-series of the first, second, and third PCs at pt.1 and pt. 38. The first PC at pt.1, which is close to the gate of the sea-dike, shows a sinusoidal tendency with irregular fluctuations, while the second and third PCs have very large irregular fluctuations as compared to the first PC (Figure 3a). The irregular fluctuations appearing on the PCs have strong correlations of above 0.9 with those appearing on the real signals of salinity, which may be directly related to the releases of freshwater. Therefore, irregular fluctuations are probably due to the high frequency of artificial freshwater discharge. On the ocean side (pt.38), irregular fluctuations were not significantly observed on all PCs since this area is far from the gate, and the clearer sinusoidal time-series appears on the first PC (Figure 3b). The second and third PCs may have some tendencies, but we do not explain them here since the present work is aiming only to find an index rather than explain whole processes appearing in the area. Overall, sinusoidal characteristics are commonly decomposed in the first PC regardless of location, which is presumably due to seasonal variability.

In order to see how T and S contribute to each PC, and how they relate to the other four variables, the eigenvectors of six variables were calculated with the values measured at all 38 points of Figure A2a. Among the 38 sets of time-series of PCs from the EOF, the results of pt.1 and pt.38 are presented in Table 2. On the closest location to the gate, pt.1, the first PC contributes 43% to the total variances, the second 32%, and the third 18%. The eigenvectors of T, DO, and TP appear to be greater than 0.5 on the first PC and S, Chl-a, and TN on the second PC. Chl-a and TN are mainly projected on the third PC, but the third PC does not contribute much to the total variances. On the ocean side, pt.38, the first PC contributes 47% to the total variances, the second 35%, and the third 11%. Here, T, S, and DO are projected onto the first PC with eigenvectors greater than 0.5, while TN and TP are on the second PC, and only Chl-a on the third PC.

The first PC is mainly affected by T and DO, regardless of location, and their sinusoidal trends are associated with seasonal variability. In addition, the first PC near the gate shows irregular and highly frequent fluctuations, which is due to the gate operation. Those fluctuations seem to reflect the contribution of TP, which is originated from the upstream of the gate. While, S contributes a lot to the second PC along with Chl-a and TN. On the ocean side, T, S, and DO show a large contribution to the first PC, reflecting the seasonality. After all, T and S show seasonal variability together in the ocean side, while near the gate, T still shows seasonal variability, but S appears close to the strong irregular variabilities. In other words, T mainly exhibits the seasonal variability along with DO, but S varies along with different variables depending on the locations (i.e., Chl-a and TN near the gate and T and DO on the ocean side). Therefore, T and S are selected as representative variables to be considered in the design of the monitoring network since they can reflect the effects of the seasonality and freshwater discharge, respectively, and also help to deduce the changes of other variables.

As a next step, we calculated the cosine angle between T and S in a three-dimensional PC space, which can be a single design index representing the whole domain of the system. A low cosine angle (i.e., near zero) means that two variables representing the eigenvectors to construct that angle originate from different sources. On the other hand, a high cosine angle (i.e., near 1) means that two variables are somewhat related and originated from similar sources.

**Figure 4:**
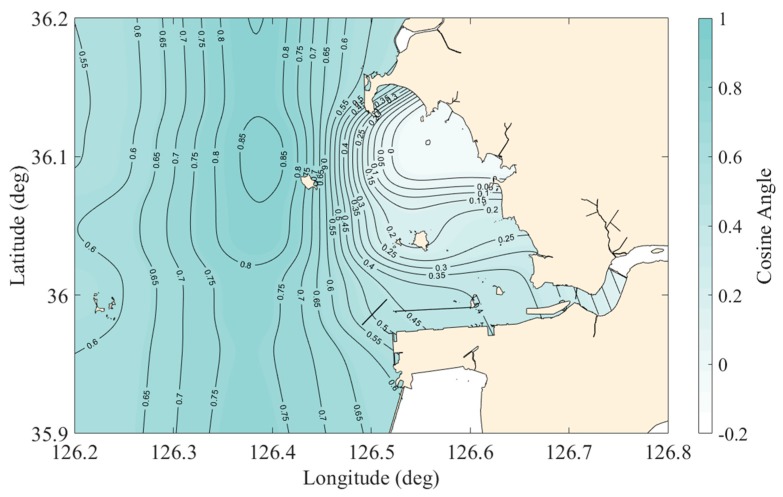
The spatial distribution of the true field of cosine angle composed of 38 points arranged at first.

Figure 4 shows the contour map of the cosine angles between the eigenvectors of T and S. The values of the cosine angle are increasing towards the open sea since T and S are simultaneously controlled by global open sea conditions such as current, solar radiation, and wind. Meanwhile, near the sea-dike, the values of cosine angles are low since, while T still responds to global open sea conditions, S reflects not only to global conditions but also to a local condition such as artificially released freshwater. The cosine angle values near pt.6 are almost 0, which means that T and S have an orthogonal (independent) tendency. This is because this area has a shallow tidal flat, so the bottom surfaces are frequently exposed to the atmosphere during low tides (tidal amplitude is around 7.5 m, [4]). Such shallow tidal flats seem to be heated up and cooled down much faster than the deep southern navigation channel (near pt.4 and 7). In addition, this area is far from the gate and, therefore, may be less affected by the freshwater discharge. 

Selecting the representative variables using EOF can extract multiple variables that represent the inherent characteristics of the coastal area. Thus, it is possible to have a complex interpretation, unlike the previous studies that used a single variable to select the monitoring points [22,23,26,29,30,31,32].

Moreover, the cosine angle has the advantage of being more reasonable because it can imply how much the representative variables have a relationship within the spatiotemporal characteristics of the other variables. Therefore, if the spatial distribution shown in Figure 4 is created and used as a design variable for optimization, a comprehensive monitoring network design that can reflect the inherent characteristics of the bay is possible.

### 3.2. Solutions for the Monitoring Array

**Figure 5:**
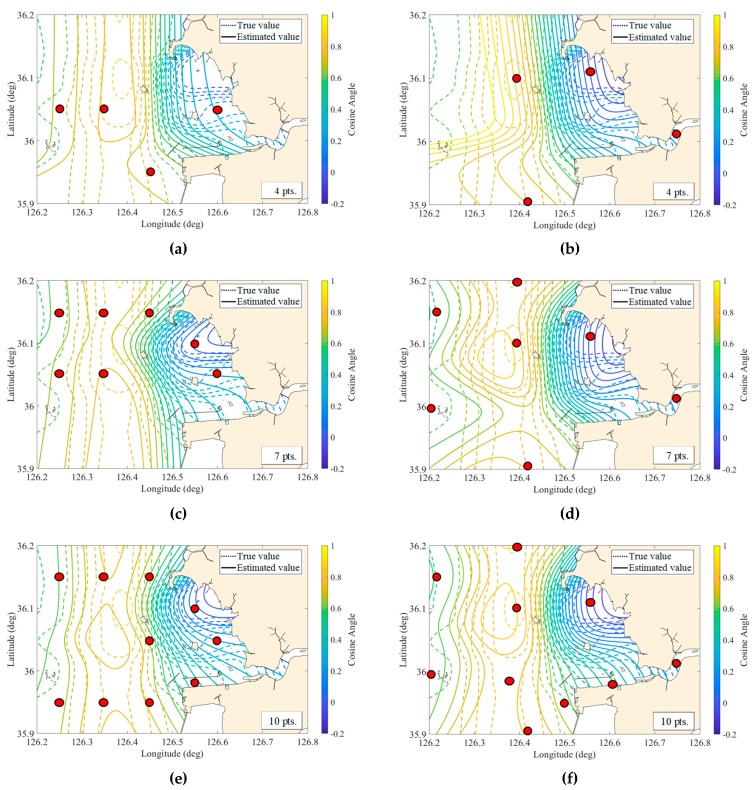
Comparison of the spatial distribution between true and estimated field reconstructed by using 4 (**a**,**b**), 7 (**c**,**d**), and 10 (**e**,**f**) points of the monitoring array, based on the quantitative optimization (left) and graphical optimization (right).

As described earlier, the objective function of quantitative optimization was composed of RMSE, and we solved it using a genetic algorithm until converging to an optimal solution. On the other hand, the graphical optimization configured the objective function by the BOA method, and the optimal array was graphically selected by using a genetic algorithm. These quantitative and graphical methods were used for searching 4 to 10 monitoring points to compare those two methods and recommend a better one. Figure 5 compares the reconstructed spatial distribution using the design index of 4, 7, and 10 monitoring points selected by quantitative and graphical optimization with the true spatial distribution. The dotted lines are the contours of the true values, and the solid lines are those of the reconstructed estimates. The spatial distribution of the graphical optimization (the panels on the right columns of the figure) reconstructs the contours more similarly to the true distribution than the quantitative optimization (the panels on the left columns of the figure). Furthermore, no matter how many searching points we want to include, the graphical optimization can find a consistent location of points (Figure 5b,d,f). However, the quantitative optimization finds different locations for each desired number of points (Figure 5a,c,e). For example, the location of 7 points searched by the graphical optimization is the same as the location of 7 points out of 10 points (Figure 5d,f). Nevertheless, 7 points found by quantitative optimization are arranged in different locations from the 10 points (Figure 5c,e).

**Figure 6:**
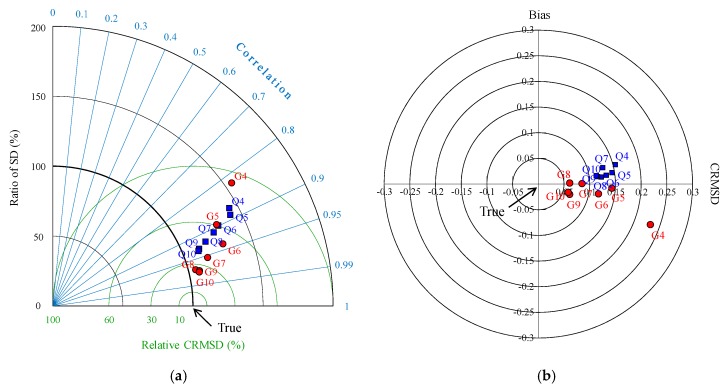
(**a**) Taylor diagram and (**b**) target diagram representing the statistics between the true and estimated spatial distribution. The abbreviation “Q” and “G” imply the quantitative and graphical optimization, respectively. The numbers after “Q” and “G” indicate the number of points selected by quantitative optimization (Q) and graphical optimization (G), respectively.

The optimal array was evaluated by the skill metrics, which plot the statistical parameters between true and estimated spatial distribution (Figure 6). First, the statistical parameters are plotted on a Taylor diagram to figure out how similar the estimated spatial distribution is to the true distribution (Figure 6a). The spatial distribution reconstructed using seven to 10 points selected by graphical optimization agrees with the true spatial distribution, with a high correlation of about 0.95 or more, and a very low CRMSD. On the other hand, if the points are selected to be six or less, the statistical points are located farther from the origin, which means poor reconstruction performance. In order to confirm how well the reconstructed distribution reproduces the variabilities of the true ones, we have identified the bias and RMSD on the target diagram (Figure 6b). The variabilities of the spatial distribution of the graphically selected points are within 0.1 of CRMSD, RMSD, and bias, for the cases with seven to 10 points. In the case of less than six points, the results do not reconstruct the true values well. On the other hand, even though nine or 10 points are selected by quantitative optimization, the results are slightly worse than seven points of graphical optimization. Therefore, quantitative optimization has a relatively poor reconstruction performance compared to the graphical optimization, except for the cases of less than four points.

**Figure 7:**
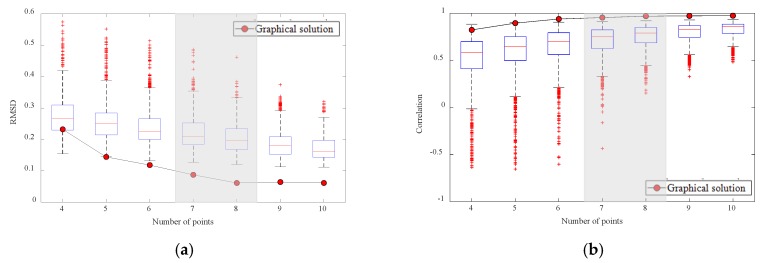
(**a**) Root mean square differences (RMSDs) and (**b**) correlation coefficient (CORs) of spatial distribution reconstructed by array of quantitative and graphical optimization.

In order to determine how many points should be selected to construct a monitoring network, RMSDs and CORs obtained by the quantitative and graphical solutions are presented in Figure 7. The box plots are the quantiles of the populations obtained by many iterations in the quantitative optimization, and the red circles are the single values found by the graphical optimization. Overall, the solutions found by the graphical optimization show better reconstruction performance, even with fewer numbers than the quantitative optimization. In addition, the graphical solution reaches a certain threshold with seven or eight points and, after reaching the threshold, converges regardless of the number of points. The solutions of quantitative optimization are different from each other depending on the number of iterations without converging on a certain value. Therefore, quantitative optimization has some statistical distributions, but graphical optimization provides a single solution without statistical distribution, since this method does not require iteration to find a solution. As a result, the graphical optimization finds the solution (i.e., representative monitoring array) with a more stable convergence to an optimal solution and less computation time [33].

The use of graphical optimization suggests several important issues in the design of the monitoring network. The first is that the inherent characteristics of the coastal area can be reflected in the objective and constrained function. For example, high spatial variability can be reflected in BOA and complex terrains and structures with a number of exterior and interior nonlinear constrained functions [19]. The second is that a complex nonlinear optimization problem can be solved with ALGA, which is known for high computational performance and global convergence [46,47]. The third is that the developed module can solve the problem of “how many” as well as “where” the monitoring points should be placed. The graphical optimization can produce arrays with consistent locations, no matter how many target monitoring points we require [33]. Therefore, implementing such an optimization module could extend the applicability of the nonlinear constraint optimization problem that can be considered for the ocean as well as for coastal areas [23].

**Figure 8:**
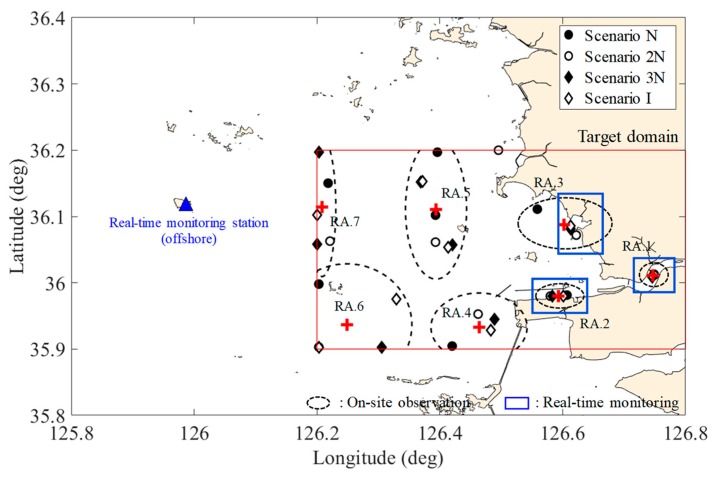
The selected points of the on-site monitoring (**red ‘+’**) and the installable area of real-time monitoring station (**blue rectangles**) in accordance with each scenario. The series of black dotted ellipses indicate maximum distances from the reference points (**red ‘+’**) corresponding to weight 1, and the blue rectangular regions are the installation area of the real-time monitoring station, which represent the temporal distribution of the local characteristics well. The blue triangle located in the outside of the target domain is the reference point of the offshore real-time monitoring station. The abbreviation “RA” imply the representative area.

The GE has considerable spatial and temporal variabilities of water quality due to the change of freshwater discharge, which can cause extreme situations [37]. Therefore, in addition to the normal case discussed earlier, and named as the scenario N here, three more scenarios were built and tested. The scenario 2N releases twice the amount of freshwater discharge than the scenario N, and the scenario 3N releases three times the amount. The scenario I reduces the amount of freshwater discharge to 50% and increases to twice the frequency of release relative to the scenario N. The numerical simulations were performed based on the scenarios, and the same method was applied to design the monitoring network for extreme events. Even though the scenarios are the functions of the amount of freshwater discharge and frequency, the representative variables are T and S, as in the scenario N. Furthermore, the trend of the first and second PCs from the EOF represents seasonality and irregular freshwater discharge and contributes to about 90% to the total time-series variance. As a result, the number of points required to reconstruct the spatial distribution by graphical optimization for three extreme scenarios is the same as for the scenario N, but their locations are slightly different from the scenario N (Figure 8).

### 3.3. Optimal Design of the Water Quality Monitoring Network

Since the on-site monitoring points selected in the four scenarios are distributed at slightly different locations, it is necessary to find a way to determine a location representing them. The time-series of data at the monitoring points of each scenario were analyzed, and the representative locations were expressed in the form of influence radius by grouping the points located near each other (Figure 8). The influence radius (black dotted ellipse) was determined by a distance-dependent weighting function of the time-series of the variable characteristics, and the center (red+) was determined by using the nonlinear least square method using Equation (7) with γ=1 and e-folding value. Since the marked points in each ellipse are solutions of each scenario, the center of the ellipsis could be regarded as a representative point that characterizes the elliptic region with the influence radius. Therefore, time-series of data acquired within the radius of influence are almost similar to the values corresponding to weight 1 from the center. Such a series of steps led to select seven representative points in GE.

In order to evaluate how well those representative points reconstruct the true distribution of all variables, the spatial distribution of each water quality variable was reconstructed and compared with the true values (Table 3). Overall, the CORs are much higher than 0.8, and the RMSD is very low in terms of their scale of mean and standard deviation. These statistical quantities mean that the representative points can reconstruct the spatial distribution to be similar to the true distribution, while expressing the spatial variability of each variable effectively. Aside from this, if the locations are selected reasonably, then with deploying even the minimum number of representative monitoring points, the spatial distribution of the six water quality variables can be relatively well reconstructed individually. Therefore, if the on-site monitoring network is designed by the framework of this study, an array, a set of representative points that have the influence radius, can be considered as an example of a good representation of GE’s spatial characteristics.

In order to determine where to install the real-time monitoring station in the representative area (i.e., area within influence radius), the signals at each area were compared and analyzed based on the reference signals of an area with the high external force or variations. The signals of the representative area (hereafter RA) 1 are assigned as a reference; RA1 is closest to the sea-dike and compared with the signals of the remaining areas. Table 4 shows the comparison of the time-series for six variables in each area with that of RA1 statistically. The CORs of the time-series show that all variables except T and DO decrease as the monitoring point gets farther away from the reference point of RA1. In addition, RMSDs increase as the points get farther away from RA1, but T and DO do not increase much relative to the magnitudes of mean and standard deviation. This is because T and DO are strongly subject to seasonal variability rather than freshwater discharge, while other variables are more significantly affected by the amount of freshwater discharge. Furthermore, in the area close to the sea-dike (i.e., RA2 and 3), the time-series of irregular freshwater discharge is reflected more than the others (i.e., RA4-7). From these results, global signals, such as seasonal variability, can be obtained in any area, while local signals, such as freshwater discharge, can only be obtained in certain areas (e.g., RA1-3). Therefore, one station must be unconditionally installed close to RA1, and other stations should be deployed near RA2 and RA3 in order to obtain the local water quality characteristics of GE.

Once a station is chosen and installed on RA1, 2, and 3 to acquire local signals, it is necessary to determine whether to install the monitoring stations in RA4, 5, 6, and 7. This is because irregular signals due to freshwater discharge can be obtained from S, Chl-a, TN, TP, in RA 1-3, while it is difficult to obtain their global signals originating from offshore characteristics. In order to determine whether to install the monitoring stations in RA4, 5, 6, and 7, the signals from the seven RAs were compared with the reference signals of an offshore observatory, which is the same location currently operated by the Korean government (Figure 8). Table 5 shows the statistical comparison of the time-series for six variables at each location with the offshore signals as the reference. 

The signals in RA4, 5, 6, and 7 are highly correlated with the offshore signals for all variables. In addition, their RMSDs show relatively low values considering the magnitudes of their means and standard deviations. However, the signals in RA1, 2, and 3 have a relatively low correlation with the signals in the offshore. In particular, S and TN cannot infer the global signals originating from the offshore with the signal in RA1, 2, and 3. Consequently, the global signals of six variables in RA4, 5, 6, and 7 are not necessarily monitored in this domain because they can be obtained from the outside of the domain and sufficiently infer all of them.

The monitoring network in Korea has been arbitrarily determined by consultation between stakeholders, engineers, and decision-makers. Moreover, no engineering studies have been conducted to design the monitoring network in the coastal area except by the authors of this study [19,33]. The marine environmental monitoring network configuration and operation plan, issued by Ministry of Maritime Affairs and Fisheries, states that “the periodic on-site observations perform in a place where it can be assumed to be representative of the area” and “real-time monitoring stations are installed and operated close to the land considering the accessibility”. After all, this is probably due to the lack of systematic procedures for designing the monitoring network [19]. As a result, a random monitoring network is composed of various national organizations (Figure 1a,b). Therefore, the monitoring network shown in Figure 8 can be a useful resource for redesigning GE’s existing monitoring network (Figure 1b) that was arbitrarily selected. In addition, the use of the present framework in other coastal regions or open oceans enables the design of more reliable monitoring networks based on an engineering basis rather than the arbitrarily designed monitoring network.

## 4. Summary and Conclusions

The coastal monitoring system, which is composed of several different series of sensors, aims to provide reliable information to forecast sea weather, sustain sound water quality, and plan for decision-making. Therefore, monitoring has been carried out to understand the inherent characteristics of the bay [19] carefully, but how the monitoring network is constructed has still not been schematically determined, but rather, it has been arbitrarily chosen. Therefore, the present study proposed a way to design an optimal monitoring network to fully reflect the spatiotemporal variability of water quality in semi-enclosed estuaries such as GE, which is a complex coastal system connected to the upstream watershed.

For designing an optimal monitoring network, instead of using ground-truth data that is not available realistically, the results from a well-validated numerical model were used to secure high-resolution assuming as ground-truth data. Such highly resolved numerical models allowed us to design a comprehensive monitoring network. With the results from the simulation, design variables were chosen to reflect the spatiotemporal characteristics of the bay adequately. As a representative design variable, the present work selected the cosine angle between the two eigenvectors of the representative variables in the three-dimensional PC space, which was determined by EOF analysis. This approach analyzed the inherent characteristics of the representative variables with other variables so that, even if the monitoring network is designed with only a variable, it can sufficiently represent the characteristics of the other variables.

The most challenging part of the present study was that we considered “where” as well as “how many” monitoring points were to be placed. Conventional quantitative optimization could determine “how many” monitoring points are needed, but the solutions converged locally so that at every trial, a consistent arrangement of solutions could not be achieved. Therefore, the graphical optimization was applied and resulted in a consistent array for each simulation once the target number of points was set without high computational cost. With the distance-dependent weighting, the interpolation functions were constrained for bounding a region to be feasible for converging the objective function to the optimal solution. After that, the array of the monitoring points could be found on the interpolated space by applying the ALGA. 

Finally, the spatiotemporal distribution, reconstructed by using the selected optimal array, was compared to the true distribution. The estimated spatial distribution was statistically evaluated by the skill metrics, on which an array of the on-site monitoring network was designed. Moreover, the installable region of the real-time monitoring station could be determined by a time-series comparison based on the reference point from which the bay’s global and local signals could be acquired. As a result, GE required a total of seven on-site monitoring points to fully represent the spatial distribution of water quality variables, and three real-time monitoring stations within the installable regions to simultaneously acquire global and local time-series characteristics.

Such a design method for finding the optimal estuarine monitoring network could be useful as a tool for strategically supporting decision-making. Besides, it is more meaningful in that the method can help not only designing the on-site monitoring array but also finding the installable regions of the real-time monitoring station that has been rarely studied so far. Such a monitoring network can reduce the cost, time, and effort for operating and managing the coastal monitoring and increase the reliability of the monitoring data [17]. Also, the design procedure of this study can strategically organize the standard framework to determine the monitoring network in a semi-enclosed estuary, as well as the lake, bay, and open ocean. Moreover, an appropriate monitoring network can secure additional advantages in improving the accuracy of hydrodynamic models for data assimilation [36,65].

## Figures and Tables

**Table 1:** The results of the EOF corresponding to the PCs for the spatial distribution. The sky blue, orange, and light green mean a group of variables that contribute to the first PC, second PC, and third PC, respectively.

Category	Principal Component	Eigenvalue	Eigenvector					
			T	S	DO	Chl-a	TN	TP
Spatial (Entire domain)	1st PC (43%)	2.56	0.26	−0.50	−0.21	0.11	0.57	0.55
	2nd PC (32%)	1.91	−0.64	−0.36	0.64	−0.10	0.23	0.01
	3rd PC (18%)	1.06	0.02	0.15	0.27	0.94	−0.07	0.12

**Table 2:** The results of the EOF corresponding to the PCs for the temporal distribution.

Category	Principal Component	Eigenvalue	Eigenvector					
			T	S	DO	Chl-a	TN	TP
Temporal (Pt.1 – near the sea-dike)	1st PC (43%)	2.59	0.58	0.10	−0.53	0.18	0.22	0.54
	2nd PC (32%)	2.20	−0.03	0.62	−0.20	0.51	−0.50	−0.25
	3rd PC (18%)	0.67	−0.14	0.10	0.39	0.66	0.62	0.05
Temporal (Pt.38 – ocean side)	1st PC (47%)	2.85	0.58	0.50	−0.52	0.31	0.04	0.23
	2nd PC (35%)	2.11	−0.10	−0.20	0.23	0.41	0.65	0.55
	3rd PC (11%)	0.67	−0.18	0.38	0.37	0.69	−0.09	−0.46

**Table 3:** Statistical quantities of the reconstructed spatial distribution for six variables.

Statistics	Water Temperature	Salinity	Dissolved Oxygen	Chlorophyll-a	Total Nitrogen	Total Phosphorus
COR	0.99	0.99	0.80	0.93	0.98	0.96
RMSD	0.07	0.46	0.06	0.24	0.06	0.00
MEAN	15.48	31.64	8.43	4.39	0.52	0.05
STD	0.45	2.68	0.10	0.60	0.25	0.01

**Table 4:** Statistical quantities of the time-series distribution for six variables at each optimal point, with representative area 1 (RA1) as a reference point.

Statistics	Water temperature						
	RA1	RA2	RA3	RA4	RA5	RA6	RA7
COR	1.00	0.99	1.00	0.96	0.95	0.88	0.90
RMSD	0.00	1.65	0.85	2.80	3.20	4.79	4.26
BIAS	0.00	0.50	−0.02	0.94	1.00	1.53	1.32
MEAN	16.38	15.88	16.40	15.44	15.38	14.85	15.06
STD	9.35	9.17	9.52	8.83	8.47	7.72	8.15
	**Salinity**						
COR	1.00	0.38	0.45	0.35	0.37	0.22	0.26
RMSD	0.00	14.36	16.87	17.53	18.15	18.71	18.75
BIAS	0.00	−13.02	−15.73	−16.37	−17.01	−17.56	−17.61
MEAN	15.47	28.48	31.20	31.84	32.48	33.03	33.08
STD	6.55	2.50	1.28	1.01	0.67	0.51	0.53
	**Dissolved Oxygen**						
COR	1.00	0.75	0.72	0.72	0.72	0.72	0.72
RMSD	0.00	1.94	2.01	2.05	2.10	2.12	2.11
BIAS	0.00	0.52	0.17	0.45	0.40	0.44	0.49
MEAN	8.82	8.31	8.65	8.38	8.43	8.38	8.33
STD	2.73	1.53	1.28	1.29	1.16	1.11	1.17
	**Chlorophyll-a**						
COR	1.00	0.82	0.80	0.77	0.73	0.66	0.69
RMSD	0.00	1.57	2.81	1.79	1.90	2.32	2.03
BIAS	0.00	−0.20	−2.02	0.37	−0.13	−0.35	0.20
MEAN	4.07	4.27	6.08	3.70	4.20	4.41	3.87
STD	2.71	2.22	3.30	1.91	2.31	2.86	2.32
	**Total Nitrogen**						
COR	1.00	0.54	0.30	0.27	0.16	0.20	0.15
RMSD	0.00	1.26	1.68	1.67	1.72	1.72	1.72
BIAS	0.00	1.10	1.51	1.50	1.56	1.56	1.56
MEAN	1.99	0.89	0.47	0.48	0.43	0.43	0.43
STD	0.74	0.27	0.05	0.05	0.04	0.04	0.04
	**Total Phosphorus**						
COR	1.00	0.84	0.59	0.62	0.62	0.66	0.64
RMSD	0.00	0.03	0.04	0.04	0.04	0.04	0.04
BIAS	0.00	0.02	0.03	0.03	0.03	0.03	0.03
MEAN	0.07	0.06	0.04	0.05	0.04	0.05	0.05
STD	0.03	0.02	0.01	0.01	0.01	0.01	0.01

**Table 5:** Statistical quantities of the time-series distribution for six variables at each optimal point with the offshore as a reference point.

Statistics	Water temperature							
	RA1	RA2	RA3	RA4	RA5	RA6	RA7	Offshore
COR	0.76	0.84	0.79	0.90	0.92	0.97	0.96	1.00
RMSD	6.58	5.51	6.49	4.52	3.98	2.49	3.09	0.00
BIAS	−2.28	−1.78	−2.30	−1.34	−1.27	−0.75	−0.96	0.00
MEAN	16.38	15.88	16.40	15.44	15.38	14.85	15.06	14.10
STD	9.35	9.17	9.52	8.83	8.47	7.72	8.15	5.99
	**Salinity**							
COR	0.16	0.29	0.10	0.65	0.56	0.89	0.85	1.00
RMSD	18.79	5.21	2.30	1.50	0.83	0.26	0.29	0.00
BIAS	17.63	4.61	1.89	1.26	0.62	0.06	0.02	0.00
MEAN	15.47	28.48	31.20	31.84	32.48	33.03	33.08	33.10
STD	6.55	2.50	1.28	1.01	0.67	0.51	0.53	0.38
	**Dissolved Oxygen**							
COR	0.72	0.97	0.97	0.99	0.99	1.00	1.00	1.00
RMSD	2.13	0.59	0.41	0.31	0.17	0.13	0.21	0.00
BIAS	−0.35	0.17	−0.17	0.10	0.05	0.10	0.15	0.00
MEAN	8.82	8.31	8.65	8.38	8.43	8.38	8.33	8.48
STD	2.73	1.53	1.28	1.29	1.16	1.11	1.17	1.04
	**Chlorophyll-a**							
COR	0.62	0.76	0.80	0.82	0.94	0.99	0.96	1.00
RMSD	3.37	2.90	2.45	3.11	2.27	1.53	2.39	0.00
BIAS	1.32	1.13	−0.69	1.69	1.19	0.98	1.53	0.00
MEAN	4.07	4.27	6.08	3.70	4.20	4.41	3.87	5.39
STD	2.71	2.22	3.30	1.91	2.31	2.86	2.32	3.93
	**Total Nitrogen**							
COR	0.28	0.40	0.75	0.80	0.94	0.98	0.95	1.00
RMSD	1.71	0.52	0.05	0.06	0.02	0.01	0.02	0.00
BIAS	−1.55	−0.46	−0.04	−0.05	0.00	0.01	0.01	0.00
MEAN	1.99	0.89	0.47	0.48	0.43	0.43	0.43	0.43
STD	0.74	0.27	0.05	0.05	0.04	0.04	0.04	0.05
	**Total Phosphorus**							
COR	0.69	0.90	0.94	0.96	0.98	0.99	0.99	1.00
RMSD	0.03	0.01	0.00	0.00	0.00	0.00	0.00	0.00
BIAS	−0.03	−0.01	0.00	0.00	0.00	0.00	0.00	0.00
MEAN	0.07	0.06	0.04	0.05	0.04	0.05	0.05	0.05
STD	0.03	0.02	0.01	0.01	0.01	0.01	0.01	0.01

## Abbreviations

The following abbreviations are used in this manuscript:

BOA	Barnes Objective Analysis
Chl-a	Chlorophyll-a
COR	Correlation
CRMSD	Centered Root Mean Square Difference
DO	Dissolved Oxygen
EOF	Empirical Orthogonal Function
G	Graphical optimization
GE	Geumgang Estuary
IOA	Index Of Agreement
OI	Optimal Interpolation
PC	Principal Component
Q	Quantitative optimization
RA	Representative Area
RE	Relative Error
RMSE	Root-Mean-Square Error
S	Salinity
SD	Standard Deviation
T	Water Temperature
TN	Total Nitrogen
TP	Total Phosphorus

## Appendix A

**Table A1:** Monthly freshwater discharge in Geumgang Estuary (Sep. 1994 – Aug. 2017).

Month		1	2	3	4	5	6	7	8	9	10	11	12	Annual
Season		Winter		Spring			Summer			Autumn			Winter	
**Discharge (10^6^ ton)**		159	160	179	228	263	468	1202	1111	795	284	200	201	5250
**Frequency**		9	9	11	13	16	19	33	33	25	15	12	11	206
**Total Time**		22	23	28	35	42	59	131	127	93	38	28	28	654
**Time/count**		2.4	2.6	2.5	2.7	2.6	3.1	4.0	3.8	3.7	2.5	2.3	2.5	3.2

**Table A2:** Calibration and validation of the numerical model [19]. The abbreviation of “SSC”, “Hs”, and “Amp” imply the suspended sediment concentration, significant wave height, and amplitude, respectively.

Variable	Parameter	Skill Score		Skill Index
		Calibration	Validation	
**Wave**	**Hs**	0.95	0.96	IOA
Tide	Semi-range	0.98	0.98	RE
	Phase-lag	1.00	0.99	
Tidal current	Amp.	0.82	0.87	RE
	Phase-lag	0.89	0.97	
SSC	-	0.65	0.64	RE
Water quality	Water temperature	0.99	0.99	IOA
	Salinity	0.57	0.85	
	Chl-a	0.67	0.67	
	TN	0.95	0.95	
	TP	0.71	0.71	
	DO	0.85	0.65	
